# 6-Amino-1,3-dimethyl-5-[(*E*)-2-(methyl­sulfan­yl)benzyl­idene­amino]­pyrimidine-2,4(1*H*,3*H*)-dione

**DOI:** 10.1107/S1600536811020903

**Published:** 2011-06-11

**Authors:** Irvin Booysen, Ismail Muhammed, Anna Soares, Thomas Gerber, Eric Hosten, Richard Betz

**Affiliations:** a University of Kwazulu-Natal, School of Chemistry, Private Bag X01, Scottsville 3209, Pietermaritzburg, South Africa; b Nelson Mandela Metropolitan University, Summerstrand Campus, Department of Chemistry, University Way, Summerstrand, PO Box 77000, Port Elizabeth 6031, South Africa

## Abstract

The title compound, C_14_H_16_N_4_O_2_S, is a Schiff base derivative of 2-(methyl­sulfan­yl)benzaldehyde. The configuration about the C=N double bond is *E*. The heterocyclic ring is essentially planar (τ = 3.1°) and makes a dihedral angle of 12.24 (7)° with the benzene ring. An intra­molecular N—H⋯S hydrogen bond is observed. In the crystal, N—H⋯O and C—H⋯O hydrogen bonds link mol­ecules into layers perpendicular to [101]. The closest distance between the centroids of two heterocyclic rings was found to be 3.5268 (8) Å.

## Related literature

For background on chelating ligands, see: Gade (1998 ▶). For the crystal structures of other Schiff bases derived from *ortho*-(thio­meth­yl)benzaldehyde, see: Yan *et al.* (2007 ▶); Baidina *et al.* (1987 ▶). For details of graph-set analysis of hydrogen bonds, see: Etter *et al.* (1990 ▶); Bernstein *et al.* (1995 ▶). For details of puckering analysis, see: Cremer & Pople (1975 ▶).
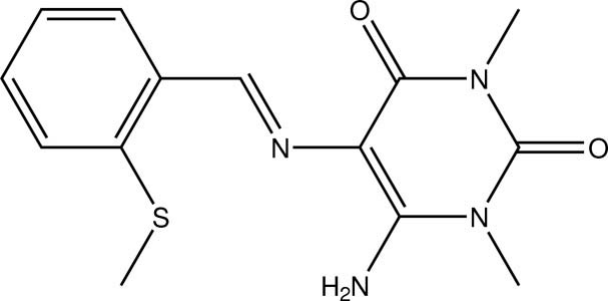


## Experimental

### 

#### Crystal data

C_14_H_16_N_4_O_2_S
*M* *_r_* = 304.37
Monoclinic, 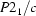

*a* = 7.9740 (2) Å
*b* = 12.4630 (3) Å
*c* = 13.9870 (3) Å
β = 94.384 (1)°
*V* = 1385.96 (6) Å^3^
*Z* = 4
Mo *K*α radiation
μ = 0.24 mm^−1^
*T* = 100 K
0.45 × 0.25 × 0.13 mm

#### Data collection

Bruker APEXII CCD diffractometer
13383 measured reflections
3441 independent reflections
2912 reflections with *I* > 2σ(*I*)
*R*_int_ = 0.025

#### Refinement

*R*[*F* ^2^ > 2σ(*F* ^2^)] = 0.036
*wR*(*F* ^2^) = 0.094
*S* = 1.09
3441 reflections
201 parameters
H atoms treated by a mixture of independent and constrained refinement
Δρ_max_ = 0.43 e Å^−3^
Δρ_min_ = −0.26 e Å^−3^

### 

Data collection: *APEX2* (Bruker, 2010 ▶); cell refinement: *SAINT* (Bruker, 2010 ▶); data reduction: *SAINT*; program(s) used to solve structure: *SIR97* (Altomare *et al.*, 1999 ▶); program(s) used to refine structure: *SHELXL97* (Sheldrick, 2008 ▶); molecular graphics: *ORTEP-3* (Farrugia, 1997 ▶) and *Mercury* (Macrae *et al.*, 2008 ▶); software used to prepare material for publication: *SHELXL97* and *PLATON* (Spek, 2009 ▶).

## Supplementary Material

Crystal structure: contains datablock(s) I, global. DOI: 10.1107/S1600536811020903/wn2434sup1.cif

Supplementary material file. DOI: 10.1107/S1600536811020903/wn2434Isup2.cdx

Structure factors: contains datablock(s) I. DOI: 10.1107/S1600536811020903/wn2434Isup3.hkl

Supplementary material file. DOI: 10.1107/S1600536811020903/wn2434Isup4.cml

Additional supplementary materials:  crystallographic information; 3D view; checkCIF report

## Figures and Tables

**Table 1:** Hydrogen-bond geometry (Å, °)

*D*—H⋯*A*	*D*—H	H⋯*A*	*D*⋯*A*	*D*—H⋯*A*
N4—H741⋯S1	0.88 (2)	2.63 (2)	3.5117 (14)	178.4 (17)
N4—H742⋯O2^i^	0.86 (2)	2.07 (2)	2.8463 (16)	150 (2)
C9—H9⋯O1^ii^	0.95	2.61	3.2807 (18)	128

Symmetry codes: (i) 
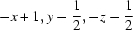
; (ii) 
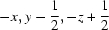
.

**Fig. 1.**
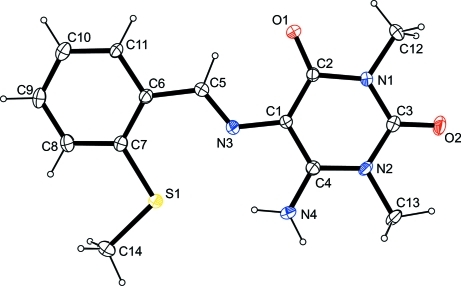
The molecular structure of the title compound, with atom labels and anisotropic displacement ellipsoids (drawn at the 50% probability level).

**Fig. 2.**
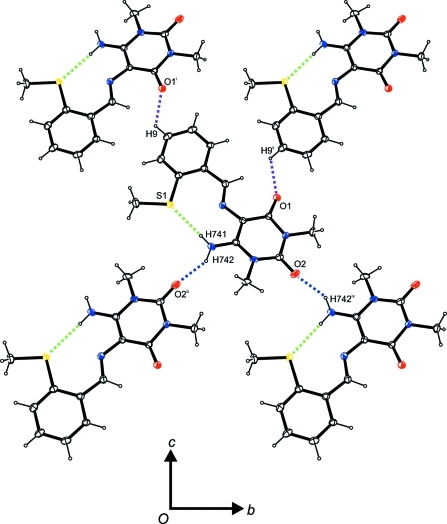
Intra- and intermolecular contacts, viewed along [-1 0 0]. Green dashed lines indicate N—H···S hydrogen bonds, blue dashed lines N—H···O hydrogen bonds and purple dashed lines C—H···O contacts. Symmetry operators: i -x, y - 1/2, -z + 1/2; ii -x, y + 1/2, -z + 1/2; iii -x + 1, y - 1/2, -z - 1/2; iv -x + 1, y + 1/2, -z - 1/2.

**Fig. 3.**
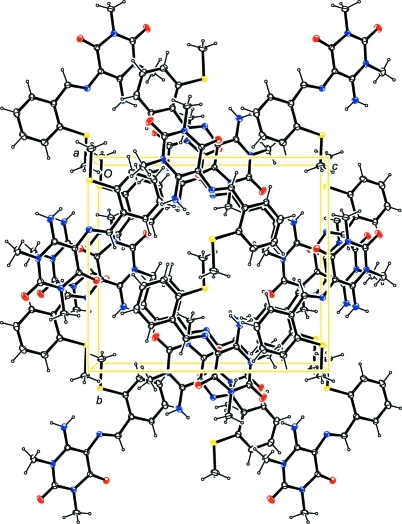
Molecular packing of the title compound, viewed along [-1 0 0] (anisotropic displacement ellipsoids drawn at 50% probability level).

## supplementary crystallographic information

### Comment

Chelate ligands have found widespread use in coordination chemistry due to the enhanced thermodynamic stability of resultant coordination compounds in relation to coordination compounds exclusively applying comparable monodentate ligands (Gade, 1998). Combining different sets of donor atoms in one chelate ligand molecule, a probe for testing and accomodating metal centers of different Lewis acidities is at hand. To enable comparative studies with envisioned coordination compounds, we determined the crystal structure of the title compound. Other crystal structures of Schiff-bases derived from *ortho*-(thiomethyl)-benzaldehyde are reported in the literature (Yan *et al.*, 2007; Baidina *et al.* 1987).

The molecule is a Schiff-base featuring an *ortho*-(thiomethyl)phenyl moiety and a 6-amino-1,3-dimethylpyrimidine-2,4(1*H*,3*H*)-dione moiety. The double-bond is (*E*)-configured. A conformational analysis of the non-aromatic six-membered ring (Cremer & Pople, 1975) fails due to the low puckering amplitude. The molecule, excluding methyl hydrogen atoms is essentially planar, the least-squares planes defined by the respective atoms of both six-membered ring systems intersecting at an angle of only 12.24 (7)° (Fig. 1).

In the crystal structure, intra- as well as intermolecular hydrogen bonds can be observed, both supported by the amino group. While the intramolecular hydrogen bond is formed to the sulfur atom of the thiomethyl group, the intermolecular hydrogen bond uses one of the ketonic oxygen atoms as acceptor. Apart from these hydrogen bonds, C—H···O contacts whose range falls by more than 0.1 Å below the sum of van-der-Waals radii of the atoms participating are apparent. These stem from the H atom bonded to the C atom in *para*-position to the Schiff-base functionality on the aromatic ring and have the second ketonic O atom as acceptor. A description of the hydrogen bonding system in terms of graph-set analysis (Etter *et al.*, 1990; Bernstein *et al.*, 1995) is possible with a *DC*^1^_1_(6) descriptor on the unitary level, whereas the C—H···O contacts necessitate a *C*^1^_1_(10) descriptor on the same level. In total, the molecules are linked in planes perpendicular to [1 0 1]. The shortest distance between the centroids of two heterocyclic rings was found to be 3.5268 (8) Å (Fig. 2). The packing of the title compound is shown in Fig. 3.

### Experimental

Equimolar amounts of *ortho*-(thiomethyl)-benzaldehyde (1.00 g, 6.57 mmol) and 5,6-diamino-1,3-dimethylpyrimidine-2,4(1*H*,3*H*)-dione (1.12 g) in 50 cm^3^ anhydrous methanol were refluxed for three hours. The reaction mixture was allowed to cool to room temperature. A yellow precipitate was isolated which was recrystallized from anhydrous acetonitrile to give yellow crystals, which were suitable for X-ray analysis.

### Refinement

H atoms bonded to Csp^2^ atoms were placed in calculated positions (C—H 0.95 Å) and were included in the refinement in the riding model approximation, with *U*(H) set to 1.2*U*_eq_(C). The H atoms of the methyl groups were allowed to rotate with a fixed angle around the C—C bond to best fit the experimental electron density (HFIX 137 in the *SHELX* program suite (Sheldrick, 2008)), with *U*(H) set to 1.5*U*_eq_(C) and C—H = 0.98 Å. Both nitrogen-bound H atoms were located in a difference Fourier map and refined freely [N—H = 0.86 (2) Å and 0.88 (2) Å].

### Crystal data

**Table:**

C_14_H_16_N_4_O_2_S	*F*(000) = 640
*M**_r_* = 304.37	*D*_x_ = 1.459 Mg m^−^^3^
Monoclinic, *P*2_1_/*c*	Mo *K*α radiation, λ = 0.71069 Å
Hall symbol: -P 2ybc	Cell parameters from 6560 reflections
*a* = 7.9740 (2) Å	θ = 2.6–28.3°
*b* = 12.4630 (3) Å	µ = 0.24 mm^−^^1^
*c* = 13.9870 (3) Å	*T* = 100 K
β = 94.384 (1)°	Rod, yellow
*V* = 1385.96 (6) Å^3^	0.45 × 0.25 × 0.13 mm
*Z* = 4	

### Data collection

**Table:**

Bruker APEXII CCD diffractometer	2912 reflections with *I* > 2σ(*I*)
Radiation source: fine-focus sealed tube	*R*_int_ = 0.025
graphite	θ_max_ = 28.3°, θ_min_ = 2.2°
φ and ω scans	*h* = −10→10
13383 measured reflections	*k* = −16→16
3441 independent reflections	*l* = −17→18

### Refinement

**Table:**

Refinement on *F*^2^	Primary atom site location: structure-invariant direct methods
Least-squares matrix: full	Secondary atom site location: difference Fourier map
*R*[*F*^2^ > 2σ(*F*^2^)] = 0.036	Hydrogen site location: inferred from neighbouring sites
*wR*(*F*^2^) = 0.094	H atoms treated by a mixture of independent and constrained refinement
*S* = 1.09	*w* = 1/[σ^2^(*F*_o_^2^) + (0.042*P*)^2^ + 0.7103*P*] where *P* = (*F*_o_^2^ + 2*F*_c_^2^)/3
3441 reflections	(Δ/σ)_max_ < 0.001
201 parameters	Δρ_max_ = 0.43 e Å^−^^3^
0 restraints	Δρ_min_ = −0.26 e Å^−^^3^

### Fractional atomic coordinates and isotropic or equivalent isotropic displacement parameters (Å^2^)

**Table:**

	*x*	*y*	*z*	*U*_iso_*/*U*_eq_	
S1	0.24479 (5)	0.11449 (3)	0.01927 (3)	0.01828 (10)	
O1	0.20013 (13)	0.57346 (8)	0.04281 (7)	0.0182 (2)	
O2	0.45091 (14)	0.64532 (9)	−0.23229 (8)	0.0219 (2)	
N1	0.32410 (15)	0.60885 (9)	−0.09573 (8)	0.0145 (2)	
N2	0.45428 (15)	0.47261 (10)	−0.17925 (8)	0.0151 (2)	
N3	0.26375 (15)	0.34072 (10)	0.01660 (8)	0.0141 (2)	
N4	0.43846 (16)	0.29401 (11)	−0.13145 (9)	0.0179 (3)	
H741	0.392 (3)	0.2487 (17)	−0.0927 (14)	0.027 (5)*	
H742	0.480 (3)	0.2713 (18)	−0.1826 (16)	0.039 (6)*	
C1	0.31118 (17)	0.42673 (11)	−0.03962 (9)	0.0133 (3)	
C2	0.27303 (17)	0.53685 (11)	−0.02460 (10)	0.0131 (3)	
C3	0.41138 (17)	0.58028 (12)	−0.17251 (10)	0.0155 (3)	
C4	0.40082 (17)	0.39688 (11)	−0.11738 (10)	0.0142 (3)	
C5	0.19312 (18)	0.35231 (11)	0.09502 (10)	0.0164 (3)	
H5	0.1728	0.4229	0.1171	0.020*	
C6	0.14235 (18)	0.26118 (12)	0.15205 (10)	0.0148 (3)	
C7	0.15496 (18)	0.15166 (12)	0.12645 (10)	0.0155 (3)	
C8	0.09458 (18)	0.07399 (12)	0.18772 (11)	0.0187 (3)	
H8	0.1007	0.0003	0.1709	0.022*	
C9	0.02593 (19)	0.10237 (13)	0.27252 (11)	0.0203 (3)	
H9	−0.0145	0.0482	0.3126	0.024*	
C10	0.01599 (19)	0.20918 (13)	0.29904 (11)	0.0201 (3)	
H10	−0.0300	0.2284	0.3573	0.024*	
C11	0.07393 (17)	0.28749 (12)	0.23952 (10)	0.0151 (3)	
H11	0.0676	0.3607	0.2579	0.018*	
C12	0.2959 (2)	0.72400 (11)	−0.08308 (11)	0.0193 (3)	
H121	0.4043	0.7604	−0.0708	0.029*	
H122	0.2275	0.7352	−0.0286	0.029*	
H123	0.2370	0.7534	−0.1413	0.029*	
C13	0.55971 (19)	0.44007 (13)	−0.25572 (10)	0.0203 (3)	
H131	0.6549	0.3976	−0.2282	0.030*	
H132	0.6017	0.5041	−0.2869	0.030*	
H133	0.4929	0.3968	−0.3032	0.030*	
C14	0.2462 (2)	−0.03061 (12)	0.02441 (12)	0.0259 (3)	
H141	0.3075	−0.0541	0.0842	0.039*	
H142	0.3016	−0.0591	−0.0304	0.039*	
H143	0.1303	−0.0572	0.0222	0.039*	

### Atomic displacement parameters (Å^2^)

**Table:**

	*U*^11^	*U*^22^	*U*^33^	*U*^12^	*U*^13^	*U*^23^
S1	0.0251 (2)	0.01325 (17)	0.01737 (18)	−0.00195 (13)	0.00745 (13)	−0.00143 (13)
O1	0.0237 (5)	0.0156 (5)	0.0163 (5)	0.0005 (4)	0.0081 (4)	−0.0011 (4)
O2	0.0257 (6)	0.0225 (6)	0.0184 (5)	−0.0023 (4)	0.0070 (4)	0.0074 (4)
N1	0.0171 (6)	0.0121 (6)	0.0145 (5)	−0.0006 (4)	0.0034 (4)	0.0017 (4)
N2	0.0155 (6)	0.0186 (6)	0.0120 (5)	−0.0005 (5)	0.0056 (4)	−0.0005 (4)
N3	0.0153 (6)	0.0126 (6)	0.0147 (6)	−0.0011 (4)	0.0016 (4)	0.0001 (4)
N4	0.0217 (6)	0.0167 (6)	0.0161 (6)	0.0006 (5)	0.0079 (5)	−0.0031 (5)
C1	0.0154 (6)	0.0117 (6)	0.0130 (6)	−0.0011 (5)	0.0027 (5)	−0.0003 (5)
C2	0.0142 (6)	0.0128 (6)	0.0125 (6)	−0.0016 (5)	0.0018 (5)	0.0005 (5)
C3	0.0145 (6)	0.0186 (7)	0.0134 (6)	−0.0017 (5)	0.0016 (5)	0.0018 (5)
C4	0.0141 (6)	0.0149 (7)	0.0135 (6)	−0.0015 (5)	0.0007 (5)	−0.0013 (5)
C5	0.0194 (7)	0.0130 (6)	0.0174 (7)	0.0008 (5)	0.0046 (5)	0.0000 (5)
C6	0.0144 (7)	0.0160 (7)	0.0143 (6)	−0.0002 (5)	0.0034 (5)	0.0007 (5)
C7	0.0148 (6)	0.0166 (7)	0.0151 (6)	−0.0012 (5)	0.0025 (5)	0.0012 (5)
C8	0.0196 (7)	0.0159 (7)	0.0207 (7)	−0.0034 (5)	0.0029 (6)	0.0032 (6)
C9	0.0187 (7)	0.0234 (8)	0.0192 (7)	−0.0042 (6)	0.0039 (6)	0.0072 (6)
C10	0.0184 (7)	0.0262 (8)	0.0162 (7)	−0.0009 (6)	0.0052 (5)	0.0031 (6)
C11	0.0161 (7)	0.0162 (7)	0.0136 (6)	0.0006 (5)	0.0058 (5)	0.0016 (5)
C12	0.0239 (8)	0.0114 (6)	0.0228 (7)	0.0009 (5)	0.0030 (6)	0.0028 (5)
C13	0.0209 (7)	0.0253 (8)	0.0160 (7)	−0.0004 (6)	0.0103 (6)	−0.0009 (6)
C14	0.0373 (9)	0.0130 (7)	0.0284 (8)	−0.0009 (6)	0.0095 (7)	−0.0030 (6)

### Geometric parameters (Å, °)

**Table:**

S1—C7	1.7719 (15)	C6—C11	1.4157 (19)
S1—C14	1.8098 (16)	C6—C7	1.417 (2)
O1—C2	1.2325 (17)	C7—C8	1.402 (2)
O2—C3	1.2231 (17)	C8—C9	1.390 (2)
N1—C3	1.3706 (18)	C8—H8	0.9500
N1—C2	1.4223 (17)	C9—C10	1.386 (2)
N1—C12	1.4655 (18)	C9—H9	0.9500
N2—C4	1.3702 (18)	C10—C11	1.385 (2)
N2—C3	1.3897 (19)	C10—H10	0.9500
N2—C13	1.4672 (17)	C11—H11	0.9500
N3—C5	1.2789 (18)	C12—H121	0.9800
N3—C1	1.3986 (18)	C12—H122	0.9800
N4—C4	1.3348 (19)	C12—H123	0.9800
N4—H741	0.88 (2)	C13—H131	0.9800
N4—H742	0.86 (2)	C13—H132	0.9800
C1—C4	1.3968 (19)	C13—H133	0.9800
C1—C2	1.4247 (19)	C14—H141	0.9800
C5—C6	1.463 (2)	C14—H142	0.9800
C5—H5	0.9500	C14—H143	0.9800
			
C7—S1—C14	103.25 (7)	C6—C7—S1	120.46 (11)
C3—N1—C2	125.02 (12)	C9—C8—C7	121.47 (14)
C3—N1—C12	116.16 (12)	C9—C8—H8	119.3
C2—N1—C12	118.59 (11)	C7—C8—H8	119.3
C4—N2—C3	122.15 (12)	C10—C9—C8	120.49 (14)
C4—N2—C13	119.56 (12)	C10—C9—H9	119.8
C3—N2—C13	118.29 (12)	C8—C9—H9	119.8
C5—N3—C1	123.47 (13)	C11—C10—C9	119.18 (14)
C4—N4—H741	114.4 (13)	C11—C10—H10	120.4
C4—N4—H742	123.0 (15)	C9—C10—H10	120.4
H741—N4—H742	120 (2)	C10—C11—C6	121.64 (14)
C4—C1—N3	114.22 (12)	C10—C11—H11	119.2
C4—C1—C2	119.94 (12)	C6—C11—H11	119.2
N3—C1—C2	125.83 (12)	N1—C12—H121	109.5
O1—C2—N1	118.60 (12)	N1—C12—H122	109.5
O1—C2—C1	126.01 (13)	H121—C12—H122	109.5
N1—C2—C1	115.39 (12)	N1—C12—H123	109.5
O2—C3—N1	122.42 (14)	H121—C12—H123	109.5
O2—C3—N2	121.06 (13)	H122—C12—H123	109.5
N1—C3—N2	116.51 (12)	N2—C13—H131	109.5
N4—C4—N2	118.96 (13)	N2—C13—H132	109.5
N4—C4—C1	120.31 (13)	H131—C13—H132	109.5
N2—C4—C1	120.71 (13)	N2—C13—H133	109.5
N3—C5—C6	122.58 (13)	H131—C13—H133	109.5
N3—C5—H5	118.7	H132—C13—H133	109.5
C6—C5—H5	118.7	S1—C14—H141	109.5
C11—C6—C7	118.76 (13)	S1—C14—H142	109.5
C11—C6—C5	115.63 (13)	H141—C14—H142	109.5
C7—C6—C5	125.61 (13)	S1—C14—H143	109.5
C8—C7—C6	118.44 (13)	H141—C14—H143	109.5
C8—C7—S1	121.10 (12)	H142—C14—H143	109.5
			
C5—N3—C1—C4	174.13 (13)	C13—N2—C4—C1	175.11 (13)
C5—N3—C1—C2	−7.2 (2)	N3—C1—C4—N4	−1.84 (19)
C3—N1—C2—O1	177.49 (13)	C2—C1—C4—N4	179.37 (13)
C12—N1—C2—O1	3.22 (19)	N3—C1—C4—N2	179.44 (12)
C3—N1—C2—C1	−3.08 (19)	C2—C1—C4—N2	0.6 (2)
C12—N1—C2—C1	−177.34 (12)	C1—N3—C5—C6	179.11 (13)
C4—C1—C2—O1	−177.39 (14)	N3—C5—C6—C11	176.78 (13)
N3—C1—C2—O1	4.0 (2)	N3—C5—C6—C7	−3.8 (2)
C4—C1—C2—N1	3.22 (19)	C11—C6—C7—C8	1.8 (2)
N3—C1—C2—N1	−175.42 (12)	C5—C6—C7—C8	−177.64 (14)
C2—N1—C3—O2	179.26 (13)	C11—C6—C7—S1	−178.33 (11)
C12—N1—C3—O2	−6.3 (2)	C5—C6—C7—S1	2.3 (2)
C2—N1—C3—N2	−1.0 (2)	C14—S1—C7—C8	−4.30 (14)
C12—N1—C3—N2	173.35 (12)	C14—S1—C7—C6	175.81 (12)
C4—N2—C3—O2	−175.03 (13)	C6—C7—C8—C9	−1.0 (2)
C13—N2—C3—O2	4.7 (2)	S1—C7—C8—C9	179.16 (11)
C4—N2—C3—N1	5.27 (19)	C7—C8—C9—C10	−0.3 (2)
C13—N2—C3—N1	−175.01 (12)	C8—C9—C10—C11	0.6 (2)
C3—N2—C4—N4	176.08 (13)	C9—C10—C11—C6	0.3 (2)
C13—N2—C4—N4	−3.6 (2)	C7—C6—C11—C10	−1.5 (2)
C3—N2—C4—C1	−5.2 (2)	C5—C6—C11—C10	177.98 (13)

### Hydrogen-bond geometry (Å, °)

**Table:**

*D*—H···*A*	*D*—H	H···*A*	*D*···*A*	*D*—H···*A*
N4—H741···S1	0.88 (2)	2.63 (2)	3.5117 (14)	178.4 (17)
N4—H742···O2^i^	0.86 (2)	2.07 (2)	2.8463 (16)	150 (2)
C9—H9···O1^ii^	0.95	2.61	3.2807 (18)	128.

Symmetry codes: (i) −*x*+1, *y*−1/2, −*z*−1/2; (ii) −*x*, *y*−1/2, −*z*+1/2.
